# Significantly improved photovoltaic performance in polymer bulk heterojunction solar cells with graphene oxide /PEDOT:PSS double decked hole transport layer

**DOI:** 10.1038/srep39555

**Published:** 2017-01-13

**Authors:** Saqib Rafique, Shahino Mah Abdullah, Muhammad Mehmood Shahid, Mohammad Omaish Ansari, Khaulah Sulaiman

**Affiliations:** 1 Low Dimensional Materials Research Centre, Department of Physics, Faculty of Science, University of Malaya, 50603, Kuala Lumpur, Malaysia; 2 Centre of Nanotechnology, King Abdulaziz University, Jeddah, 21589, Saudi Arabia; 3 International Institute of Advanced Islamic Studies (IAIS) Malaysia, Jalan Ilmu, Off Jalan Universiti, 59100, Kuala Lumpur, Malaysia

## Abstract

This work demonstrates the high performance graphene oxide (GO)/PEDOT:PSS doubled decked hole transport layer (HTL) in the PCDTBT:PC_71_BM based bulk heterojunction organic photovoltaic device. The devices were tested on merits of their power conversion efficiency (PCE), reproducibility, stability and further compared with the devices with individual GO or PEDOT:PSS HTLs. Solar cells employing GO/PEDOT:PSS HTL yielded a PCE of 4.28% as compared to either of individual GO or PEDOT:PSS HTLs where they demonstrated PCEs of 2.77 and 3.57%, respectively. In case of single GO HTL, an inhomogeneous coating of ITO caused the poor performance whereas PEDOT:PSS is known to be hygroscopic and acidic which upon direct contact with ITO reduced the device performance. The improvement in the photovoltaic performance is mainly ascribed to the increased charge carriers mobility, short circuit current, open circuit voltage, fill factor, and decreased series resistance. The well matched work function of GO and PEDOT:PSS is likely to facilitate the charge transportation and an overall reduction in the series resistance. Moreover, GO could effectively block the electrons due to its large band-gap of ~3.6 eV, leading to an increased shunt resistance. In addition, we also observed the improvement in the reproducibility and stability.

Organic photovoltaic devices (OPVs) employing donor-acceptor bulk heterojunction (BHJ) structure are considered promising next generation solar cells due to their advantages over traditional counterparts, including lower costs, increased flexibility, lighter weight plus solution processed roll to roll (R2R) production compatibility^1^,^2^,^3^. Although this class of solar cells has seen significant progress, further development in both efficiency as well as stability are still needed for their widespread commercial applications^4^. During recent years, considerable research has been focused on interfacial engineering of OPVs, in particular, on the introduction of an interfacial layer between indium tin oxide (ITO) anode and a photo-active layer that could facilitate the transportation of holes, blocking of electrons and reduce the charge recombination and leakage^5^,^6^. This layer is often termed as hole transport layer (HTL).

Poly (3,4-ethylenedioxythiophene): Poly (styrenesulfonate) (PEDOT:PSS) is regarded as state of the art HTL which is being used as a standard material for BHJ OPVs because of its high work function, easy solution process-ability, high conductivity and high optical transmittance^7^,^8^. However, owing to the highly acidic and hygroscopic nature of PEDOT:PSS, it favours the device degradation in number of ways^9^,^10^. The chemical interaction between PEDOT:PSS and ITO causes the corrosion of ITO which gives rise to severe instability in device performance^11^. In addition, it absorbs the oxygen and water from the air which further penetrate to subsequent layers to eventually reduce the device performance^3^. Therefore, research has been focused to either replace or improve PEDOT:PSS by introducing inorganic semiconductors such as V_2_O_5_^12^,^13^, NiO^14^, WO_3_^15^ or MoO_3_^16^, among others, to address the reliability issues related to PEDOT:PSS. However, deposition of these oxide materials normally involves intensive costs related to high vacuum techniques which are incompatible with the large scale R2R OPV production.

In this context, solution processed aqueous dispersion of graphene oxide (GO) has been recently used by several groups as an HTL material for ITO anode^17^,^18^,^19^. GO is the derivative of one atom thick graphene comprises of hydroxyl (OH) and epoxy group on its basal plane and carboxyl groups (COOH) at the edge^20^. GO, in aqueous dispersion, exhibits a unique heterogeneous electronic structure due to the presence of mixed sp^2^ and sp^3^ hybridizations^21^. However, it lacks good Ohmic contact due to its insulating properties^22^ Moreover, it is difficult to obtain the full coverage coating of GO at a time. Therefore, recently combination of GO and PEDOT:PSS have been reported to effectively work as an HTL in OPVs. It is reported that use of a thin layer of GO underneath PEDOT:PSS can effectively prevent corrosion of ITO and its further diffusion into the photoactive layer^23^. Lee, Da-Young *et al*.^24^, in their recent work on planar heterojunction perovskite solar cells, used GO/PEDOT:PSS HTL structure to obtain a stable device with power conversion efficiency (PCE) of 9.74%. Similarly, Yu, Jae Choul *et al*.^22^ demonstrated highly efficient polymer light emitting diodes (PLEDs) and OPVs with GO and PEDOT:PSS composite layer as an HTL. Y. Park *et al*.^25^, used GO/PEDOT: PSS bi-layer HTL in P3HT:PCBM based BHJ OPVs and demonstrated a PCE of 3.53%. In this context, we used poly[N-9′-heptadecanyl-2,7-carbazole-alt-5,5-(4′,7′-di-2-thienyl-2′,1′,3′benzothiadiazole)] (PCDTBT): (6,6)-Phenyl C_71_ butyric acid methyl ester (PC_71_BM) photoactive blend layer in BHJ OPVs during this study. PCDTBT, as donor polymer, is expected to yield high efficiency and photo-current generation due to its faster charge carrier generation capability and different recombination dynamics as compared to P3HT^26^,^27^,^28^. In addition, PCDTBT work function (WF) matches well with that of GO and PEDOT:PSS as compared to P3HT.

In the present study, findings suggest the enhanced efficiency of PCDTBT:PC_71_BM based OPVs, using a solution processed GO/PEDOT:PSS double decked layer as an HTL. It is suggested that combination of GO/PEDOT:PSS as an HTL may complement the shortcomings of either of individual materials. GO in combination with PEDOT:PSS as an HTL exhibited a high efficiency and stability as compared to either of single PEDOT:PSS or GO HTLs. This study investigated the electrical, optical, chemical and morphological properties and their effects on the performance of the OPVs.

## Experimental Methods

### Materials

PEDOT:PSS aqueous suspension (PH1000) was purchased from H.C. Starck and used as received. Both PCDTBT and PC_71_BM have been purchased from Lumtec, Taiwan. Pre-patterned ITO-coated glass substrates with a sheet resistance of 15 Ω per square were purchased from Ossila, UK. Graphite flakes has been purchased from Asbury Inc. (USA). Potassium permanganate (KMnO_4_, >99%), sulphuric acid (H_2_SO_4_, 98%), phosphoric acid (H_3_PO_4_, 98%), and hydrochloric acid (HCl, 35%) for GO synthesis, were obtained from R & M Chemicals. All other necessary chemicals such as chloroform etc. were purchased commercially and used as received without further purification.

### Synthesis of GO

In the present study, GO was synthesised following a simplified hummers method^29^. Namely, graphite flakes, H_3_PO_4_, H_2_SO_4_, and KMnO_4_ were mixed in an appropriate amount at room temperature by using a magnetic stirrer. The mixture was kept on stirring for about 72 h so that complete oxidation of the graphite could be achieved. After oxidation of graphite, H_2_O_2_ solution along with ice was used to stop the oxidation. The synthesised GO was subjected to washing for three times with 1 M of HCl aqueous suspension and several times with de-ionised (DI) water to achieve a neutral pH. During washing process with DI water, the exfoliation of GO was experienced, which resulted in formation of thick brown GO solution and finally followed by the emergence of the GO gel. The concentration of obtained GO gel was determined for further studies.

### Device fabrication procedure

Pre-patterned ITO coated glass substrates were cleaned with consecutive ultrasonic agitation in acetone, isopropyl alcohol (IPA) and (DI) water for 20 minutes each. The substrates after drying with nitrogen stream were subjected to oxygen plasma treatment to form a hydrophilic surface state. The GO aqueous solution was prepared at the concentration of 1 mg/ml in DI water, while, PEDOT:PSS solution was filtered by using 0.45 μm PTFE filters (Whatman, Germany). The doubled decked (GO/PEDOT:PSS) HTLs were deposited by sequential spin coating of GO and PEDOT:PSS at 6000 rpm for 60 seconds onto the ITO coated substrates and post-annealed at 150 °C for 5 min in ambient room conditions. For the performance comparison, individual GO and PEDOT:PSS HTLs were also deposited and annealed at the same conditions. To perform further fabrication steps, all the materials and substrates were transferred to nitrogen (N_2_) filled glove box. To fabricate the photo- active layer, both PCDTBT (donor) and PC_71_BM (acceptor) were dissolved in chloroform at the concentration of 10 mg/ml by vigorous stirring overnight and further mixed at the ratio of 1: 4, respectively. The prepared blend was first filtered by 0.25 μm PTFE filters and then spun coated at an optimised speed of 2000 rpm for 60 s onto PEDOT:PSS, GO and GO/PEDOT:PSS HTLs. Next, aluminium (Al) electrodes were thermally evaporated onto the active layer through shadow masks under vacuum with the pressure of 10^−6^ Torr. Thereafter, the fabricated devices were encapsulated with glass covering the active area by using UV-curable epoxy for the characterisations in the air.

### Instrumentations

The surface morphologies of the all three types of HTLs were analysed by atomic force microscopy (AFM) using tapping mode of Agilent Technologies 5500 Scanning Probe Microscope. Cross-section images were taken by field emission scanning electrons microscopy (Hitachi, SU8220 Scanning Electron Microscope). Raman spectra of all types of HTLs were measured by a DXR Raman Microscope (Thermo Scientific, USA), by using green light excitation (532 nm) laser source with 6 mW power. Optical properties were measured by a Perkin Elmer UV-visible diffuse reflectance spectrophotometer (Lambda 650) in the range of 250–800 nm. XPS analysis of prepared GO was carried out by PHI 5000 Versa Probe Scanning ESCA Microprobe (PHI 5000 Versa Probe II, USA), equipped with monochromatic Al-Kα (hν = 1486.6 eV) X-ray source. We performed curve fittings for core level spectrum by using Multipack software (VERSION 9, ULVAC-PHI, Inc. Japan) which allows the deconvolution of each spectrum into the individual fitting of mixed Gaussian-Lorentzian components.

Current density-voltage (*J-V*) characteristics of OPVs were measured using a Keithley 236 (Keithley Co.) source measurement unit. Photovoltaic performance was measured by using an Air Mass 1.5 Global (AM 1.5 G) solar simulator with an irradiation intensity of 100 mW/cm^2^. The light intensity was calibrated using a Newport power meter 1918-R with calibrated Si-detector 818-UV.

## Results and Discussion

**Figure 1:**
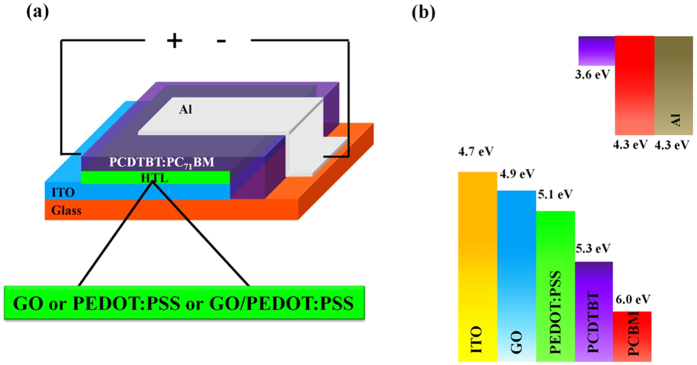
(**a**) Schematic illustration of the BHJ OPVs with GO, PEDOT:PSS and GO/PEDOT:PSS HTLs and (**b**) The energy band diagram showing the energy levels of all the materials used in OPVs of current study.

In the present study, the BHJ OPVs were fabricated with single GO, PEDOT:PSS and double decked GO/PEDOT:PSS as HTLs. GO possesses several unique advantages, including its tuneable energy levels, facile solution processed, low cost synthesis, its two-dimensional structure and easy functionalization^30^. However, it is essential to fully cover the ITO surface with a uniform and very thin layer to achieve an optimum performance with a GO HTL. Moreover, post deposition annealing is also recommended to remove the oxygen function groups and consequently the electrical properties of GO could be improved^17^. It is difficult to deposit highly uniform thin layer of GO with full coverage of ITO surface. As a result, poor holes extraction to ITO anode can be expected because of the direct contact of BHJ photo-active layer with the ITO at the uncovered regions. In addition, non-uniform surface coverage by GO may also lead to inhomogeneous electrical properties yielding a poor reliability of the device performance^24^. Similarly, standard PEDOT:PSS HTL also exhibits severe stability issues. To address these point, we used low temperature (150 °C) solution processed approach to fabricate BHJ OPVs with sequential spin coating of GO and PEDOT:PSS and compared with that of individual GO or PEDOT:PSS HTLs on merits of their efficiency, reproducibility and stability. The schematic of the current work is presented in Fig. 1.

### Spectroscopic characterisations

**Figure 2:**
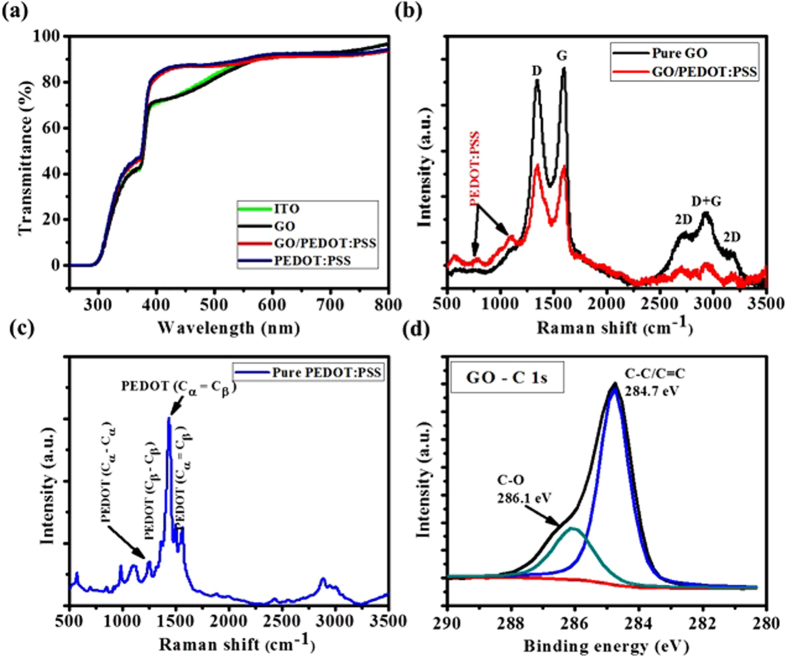
(**a**) Transmittance of GO, PEDOT:PSS and GO/PEDOT:PSS films deposited on ITO coated substrate and that of bare ITO substrate. (**b**) Raman spectra for GO, GO/PEDOT:PSS and (**c**) PEDOT:PSS HTLs. (**d**) Deconvoluted XPS C 1s spectrum of GO.

The optical and structural properties of each HTL deposited on ITO coated glass substrates have been investigated prior to device fabrication. The transmittance spectra of all three types of HTLs along with bare ITO are shown in Fig. 2a. These layers show high transmittance in the overall wavelength range of more than 87%. The optical transparency of HTLs is very important in order to absorb maximum light by photo-active layer. It can be observed that the transmittance spectra of the ITO/PEDOT:PSS and ITO/GO/PEDOT:PSS is almost 15% higher compared with the transmittance of pristine ITO and ITO/GO in the region of ~400–500 nm. However, no pronounced change in transmittance spectra for any of the HTLs is observed in the overall wavelength region except the aforementioned wavelength range. The observed difference of transmittance in the ~400–500 nm region could potentially bring a significant effect on the photo-generated current (*J*_*sc*_) of the device and hence the better efficiency is expected in case of ITO/PEDOT:PSS and ITO/GO/PEDOT:PSS, in good agreement with the photovoltaic studies of the corresponding devices.

Raman spectroscopy is the most commonly used non-destructive technique to analyse the quality and structure of the carbon based materials, in particular, it is being used to investigate the defects and ordered and disordered structures of graphene^31^. Raman spectrum was collected for GO/PEDOT:PSS double decked structure as shown in Fig. 2b. For comparison, we also collected the spectrum of single GO and PEDOT:PSS HTLs. Raman spectrum for single GO HTL shows both D and G bands appearing at 1350 and 1600 cm^−1^, respectively. It is well known that D band appearing in the range of 1200 to 1500 cm^−1^ corresponds to structural defects and partially disordered structures of the sp^2^ domains, whereas, G band appearing from 1500 to 1600 cm^−1^ is associated with E_2g_–vibration mode of sp^2^ carbon^31^,^32^. The wide band towards high frequency end of the spectrum features three peaks at around 2720, 2930 and 3190 cm^−1^. The peak at 2790 cm^−1^ is corresponding to an overtone of D band and attributed as 2D band, whereas, the peak at 2930 cm^−1^ arise from contribution of both D and G bands and often termed as D + G band^33^,^34^. The shoulder peak at ~3190 cm^−1^ also ascribes to an overtone of D band (2D). The Raman spectrum for GO/PEDOT:PSS films also illustrated D and G bands but with significantly decreased intensity of the peaks as shown in Fig. 2b. In addition, the spectrum is featured with some bands from PEDOT:PSS polymer in low frequency range between 500 to 1100 cm^−1^ which confirms the method efficacy of double decked layer fabrication. The GO/PEDOT:PSS HTL also features the 2D, D + G and 2D bands at 2700, 2930 and 3170 cm^−1^ but with significantly low intensity as compared to pure GO films. The Raman spectrum for PEDOT:PSS HTL shown in Fig. 2c exhibits Raman finger prints for PEDOT and PSS. Most of the peaks are attributed to PEDOT and negligible contribution of PSS is observed in the spectrum, in good agreement with previously reported data^1^,^35^. A strong vibrational band observed at 1440 cm^−1^ is attributed to PEDOT and related to symmetric Cα = C_β_ (−O) stretching mode. In addition, the following bands are related to PEDOT vibrational modes such as: 1562 cm^−1^ is ascribed to Cα = C_β_ asymmetrical stretching, 1364 cm^−1^ to C_β−_ C_β_ stretching deformations and 1255 cm^−1^ to C_α−_ C_α_ inter-ring stretching vibrations^36^,^37^. The peaks at 986 and 573 cm^−1^ correspond to the oxyethylene ring deformation^38^.

The core level XPS C 1s spectrum of the GO presented in Fig. 2d is decomposed into two components, the sharp and high intensity peak at 284.7 eV is ascribed to sp^2^ carbon aromatic rings (C-C/C=C) and relatively low intensity peak at 286.1 eV corresponds to C-O bond^31^,^39^. These peaks confirm the presence of carbon atoms in different functional groups, i.e. the non- oxygenated rings and oxygen related functional groups^40^,^41^.

### Morphological study of HTLs

**Figure 3:**
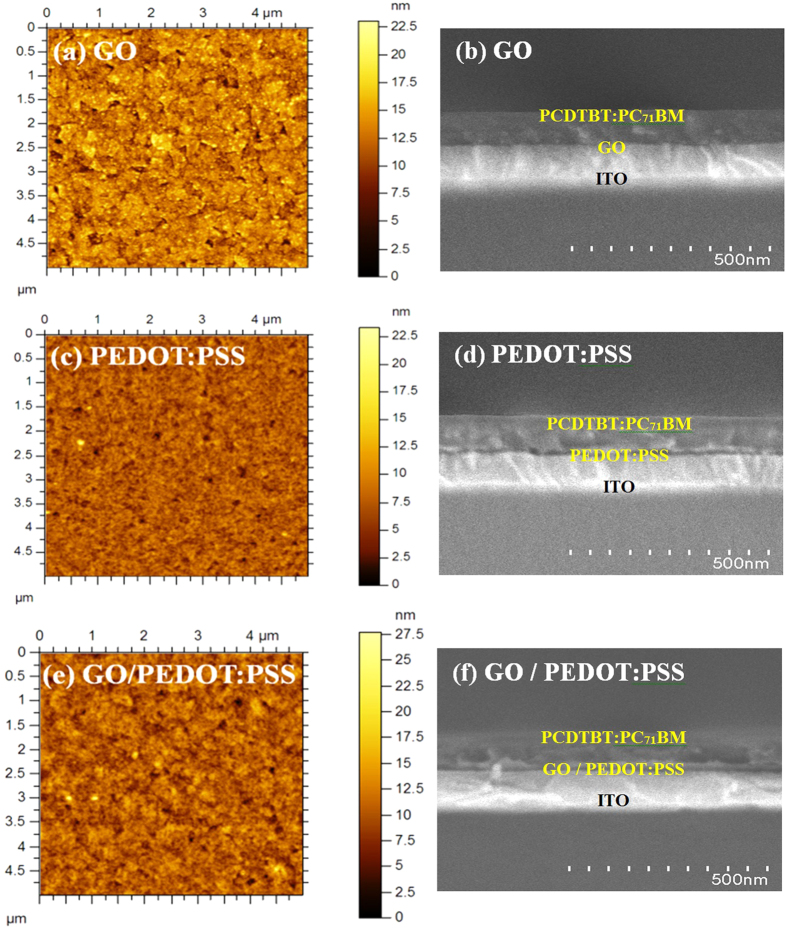
The AFM surface topography images and SEM cross-sectional images with complete device, of (**a,b**) GO (**c,d**) PEDOT:PSS and (**e,f**) GO/PEDOT:PSS HTLs.

Film morphology of an HTL significantly influences the electrical properties of the device, in particular, the series (R_s_) and shunt resistance (R_sh_). In general, a smooth and fully-covered HTL morphology may induce a higher R_sh_ and low R_s_ which is highly desirable to enhance the performance of OPVs^17^. It is therefore vital to control the morphology of the HTLs. In the present study, we observed the AFM topography images of the all three HTLs on ITO as shown in Fig. 3. The root mean square (RMS) roughness values of GO, PEDOT:PSS and GO/PEDOT:PSS films in an area of 5 μm × 5 μm were calculated to be 2.88, 1.56 and 1.99 nm, respectively. As compared to the recently reported results (3.2 nm) for spin coated GO films^24^, the RMS roughness of our samples is comparatively improved but it is still significantly higher than PEDOT:PSS HTL. The single GO HTL is inhomogeneous with overlapping GO flakes across the surface as visible in AFM image (Fig. 3a) and could not cover the ITO surface with high uniformity. Consequently, the non-uniform and overlapping GO flakes could suppress the transportation of holes while the uncovered areas may lead to direct contact of ITO and photo-active layer and hence the performance of OPVs with GO as HTLs could significantly reduce^20^. By applying PEDOT:PSS on GO (GO/PEDOT:PSS HTL), RMS roughness reduced to 1.99 nm (Fig. 3e), comparable with single PEDOT:PSS, (Fig. 3c) which indicate that deposition of PEDOT:PSS on GO results in smoothening of the irregular GO surface.

Figure 3 shows cross-sectional SEM images of the solar cells fabricated with each of the GO, PEDOT:PSS and GO/PEDOT:PSS HTLs. The thickness of PCDTBT:PC_71_BM were observed to be uniform (approx. 75 nm) regardless of the HTLs. The single GO film was very thin (around 1–3 nm) to distinguish as shown in Fig. 3b. The thickness of both PEDOT:PSS (Fig. 3d) and GO/PEDOT:PSS (Fig. 3f) HTLs was approximately the same and calculated to be around 30 nm.

### Photovoltaic characteristics

**Figure 4:**
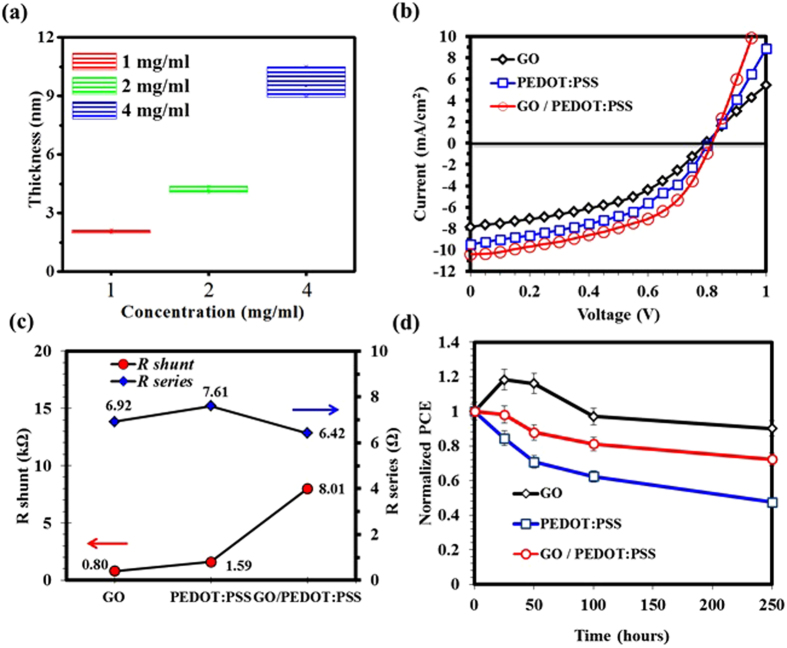
(**a**) Thickness *vs* concentration plots for GO. (**b**) J-V characteristics of OPVs with GO, PEDOT:PSS and GO/PEDOT:PSS as an HTL. (**c**) R_sh_ and R_s_ calculated from J-V curves under illumination conditions. (**d**) Stability test of OPVs over 250 h.

In order to analyse the photovoltaic characteristics, it is important to explain the role of HTL during the OPVs operation. In normal architecture BHJ OPVs, the photo-active layer is irradiated with solar light via ITO/HTL bottom electrode side, while the active layer absorbs the solar light (photons) and generates electrons- holes pairs, the so-called excitons. Further, these excitons dissociate into the electrons in the lowest unoccupied molecular orbital (LUMO) and holes in the highest occupied molecular orbital (HOMO), at the donor-acceptor interface. Therefore, the HOMO level of the donor polymer should match well with the work-function of HTL to facilitate the transportation of holes through HTLs to the anode^19^. In this context, PCDTBT with the HOMO of 5.3 eV was utilised along with the GO/PEDOT:PSS HTL. The device with PCDTBT as donor polymer provides better holes extraction since energy levels from PCDTBT/PEDOT:PSS/GO/ITO provide good ascending steps for the holes to hop. The energy level diagram for each element used in this study and the device structure are presented in Fig. 1. Reference devices with only GO and PEDOT:PSS as an HTL were also fabricated for comparison. In addition, the performance of GO is somehow thickness dependent and one can achieve an optimum performance with the GO layer of 1–3 nm^42^. Therefore, we optimised the thickness and concentration of GO, and with the spinning conditions described in the experimental section we were able to achieve a layer of approximately 1–3 nm as shown in Fig. 4a.

Figure 4b shows the typical current density-voltage (*J-V*) characteristics under illumination of the PCDTBT:PC_71_BM OPV devices with GO, PEDOT:PSS and GO/PEDOT:PSS as the HTLs, and the corresponding extracted device parameters are summarised in Table 1. As shown in *J-V* curves, the devices using the GO/PEDOT:PSS double decked HTL showed superior performance as compared to the individual GO or PEDOT:PSS HTLs with an open circuit voltage (V_oc_) of 0.82 V, a short-circuit current (J_sc_) of 10.44 mA/cm^2^, a fill factor (FF) of 0.50 and a power conversion efficiency (*η*) of 4.28%. In comparison, the devices with only PEDOT:PSS as an HTL exhibited a relatively low performance with V_oc_ of 0.80 V, a J_sc_ of 9.49 mA/cm^2^, a FF of 0.47 and an *η* of 3.57%. Whereas, the device with single GO exhibited poor performance with a V_oc_ of 0.80 V, a J_sc_ of 7.90 mA/cm^2^, a FF of 0.44 and an *η* of 2.77%. Relatively poor performance of the devices with individual GO HTL is mainly attributed to inhomogeneous GO layer with high surface roughness that lead to suppression and an inefficient transportation of holes. As a result, the device efficiency is significantly reduced. For the devices based on GO/PEDOT:PSS HTL, the improvement is attributable to high J_sc_, FF and V_oc_ values as compared to either of GO or PEDOT:PSS HTLs. Additionally, the GO/PEDOT:PSS HTL also exhibited reduced R_s_ than GO or PEDOT:PSS single HTLs, as shown in Fig. 4c. Relatively low R_s_ value in case of GO/PEDOT:PSS HTL suggest the better charge transportation ability of the double decked structure as compared to single GO and PEDOT:PSS based devices. As discussed above and presented in the energy diagram in Fig. 1, the WF of GO (4.9 eV) matches well with PEDOT:PSS (5.1 eV) which likely results in an efficient charge extraction and transportation to ITO. Moreover, GO could effectively block the electrons owing to its large band-gap of ~3.6 eV^44^.

Since long term stability of the OPVs is one of the most important factors for their widespread commercialisation, therefore, we explored the long-term operational stability of all type of HTLs. Figure 4d shows the decay in PCE as a function of exposure time in ambient atmosphere. The devices with single GO HTLs or with GO/PEDOT:PSS HTLs showed better stability as compared to single PEDOT:PSS HTLs in which the efficiency decreased to more than 50% of initial value. The instability in the PEDOT:PSS HTL is attributed to corrosion of indium due to acidic nature of PEDOT:PSS with high pH value^43^. With the passage of time indium diffuses into the HTL and further to active layer which causes severe damage to the device performance. The improved stability in case of single GO/PEDOT:PSS HTL is expected because a thin layer of GO underneath PEDOT:PSS serves as a barrier against the direct contact of PEDOT:PSS with ITO surface.

**Figure 5:**
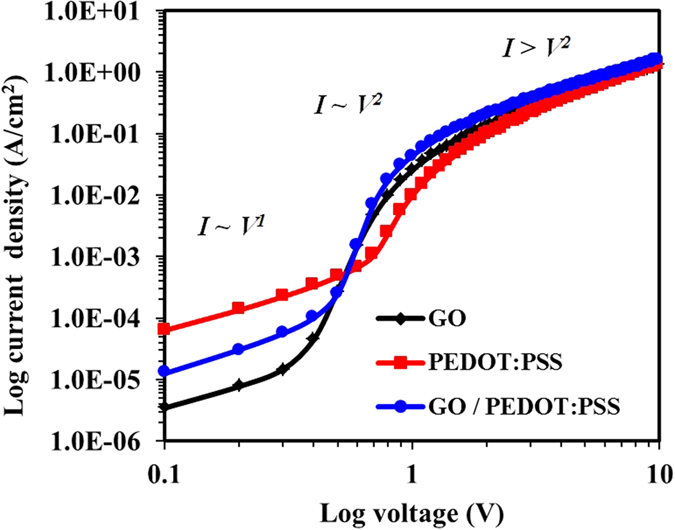
Double logarithmic characteristic (Log J vs. log V) exhibiting effective charge carrier mobility of PCDTBT:PC_71_BM based OPVs with GO, PEDOT:PSS and GO/PEDOT:PSS as the HTLs.

The effective carrier mobility or the so-called ambipolar mobility for all three devices was then determined by space-charge-limited-current (SLCL) method under positive voltage up to 10 V in dark. Figure 5 shows *log J* vs. *log V*, several conduction regimes have been identified from the plots based on their gradient values, such as: *I* ~ *V^1^* with slope 1 is an Ohmic regime, *I* ~ *V^2^* with slope 2 is an SCLC regime, while *I* > *V^2^* with slope >2 is a trap regime. The ambipolar mobility has been calculated from the SCLC regime by the following equation:


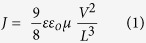


where *J* is the current density within the conduction regime, *ε* is the relative dielectric constant, *ε*_*o*_ is the vacuum permittivity, *L* is the active layer thickness, *V* the voltage within the conduction regime and *μ* represents the mobility^44^. Figure 5 and Table 1 show the change of ambipolar mobility for the devices with GO, PEDOT:PSS and GO/PEDOT:PSS HTLs. The ambipolar mobility increased from 3.78 × 10^−4 ^cm^2^ V^−1^ s^−1^ in case of PEDOT:PSS HTL to 4.04 × 10^−4 ^cm^2^ V^−1^ s^−1^ for single GO HTL and finally to 7.47 × 10^−4 ^cm^2^ V^−1^ s^−1^ for the device with GO/PEDOT:PSS double decked HTL, which is almost double of the either of the individual GO or PEDOT:PSS HTLs, and in good agreement with the PCEs of corresponding devices. A quantitative comparison of ambipolar mobility of the OSCs with GO, PEDOT:PSS and GO/PEDOT:PSS HTLs confirmed that use of GO/PEDOT:PSS layer promoted the charge carriers transportation and extraction, particularly the hole mobility, resulting in optimized photovoltaic performance^22^. Since the only difference in the device architecture is the utilization of different HTLs therefore the increase in the accumulative charge carrier mobility is predominately due to the increased hole mobility using a GO/PEDOT:PSS double decked layer that can lead to balanced charge carrier transportation with an electron-blocking ability and a reduction of the suppression between the HTL and the active layer^22^. Thus, OSCs with high PCEs were realized using GO/PEDOT:PSS double decked layer.

**Figure 6:**
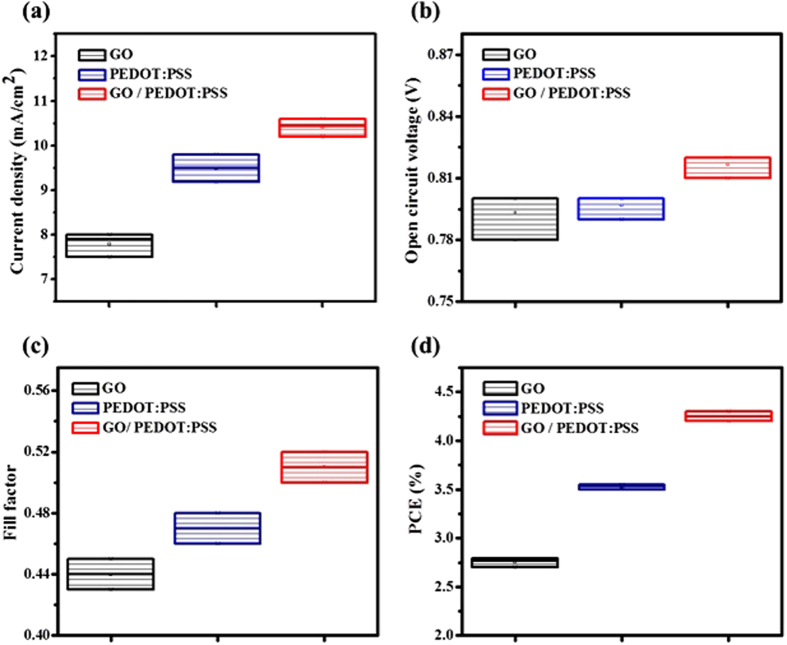
Photovoltaic performance parameters including (**a**) J_sc_, (**b**) V_oc_, (**c**) FF and (**d**) PCEs (%) of PCDTBT:PC_71_BM based OPVs with GO, PEDOT:PSS and GO/PEDOT:PSS as the HTLs.

In order to confirm the reproducibility and reliability of the GO/PEDOT:PSS HTLs, we recorded the important photovoltaic parameters and presented in Fig. 6. The GO/PEDOT:PSS HTL exhibited fairly good reproducibility with minor variations in V_oc_, J_sc_ and PCE. On the contrary, the device with GO HTL showed comparatively wide variations in V_oc_. However, a relatively wider variation is observed in FF of all HTLs which could be attributed to the absence of electron transport layer (ETL) in our device structure.

The selection of double decked GO/PEDOT:PSS HTL played a vital role in the improved device efficiency and stability. It has been reported that use of either of individual GO or PEDOT:PSS as HTLs in OPVs may cause severe instability issues at the anode interface^24^,^45^. The hygroscopic and acidic PEDOT:PSS aqueous suspension could react with both the ITO as well as the photoactive layer. Similarly, single and ultrathin GO layer could cause a non-uniform coating on ITO which would provide a direct contact of ITO and photoactive layer at the non-covered regions and drastically reduces the device performance. Interestingly, the combination of GO and PEDOT:PSS in a double decked structure is a compatible solution to compliment the drawbacks of both individual materials. To the best of our knowledge, there is no work reported on photovoltaic performance, reproducibility and stability of PCDTBT:PC_71_BM based devices with GO/PEDOT:PSS double decked layer.

## Conclusions

In summary, the performance, reproducibility and stability of GO/PEDOT:PSS double decked HTL in PCDTBT:PC_71_BM based OPVs is reported. The GO/PEDOT:PSS is a promising candidate to replace conventional PEDOT:PSS or single GO HTLs by complimenting the drawbacks of both individual materials. Our GO/PEDOT:PSS HTL retained its efficiency as well as reproducibility yielding a highly stable device. It demonstrated a J_sc_ = 10.44 mA/cm^2^, V_oc_ = 0.82 V, FF = 0.50, and PCE = 4.28%. A well matched work function of GO/PEDOT:PSS = 4.9 eV/5.1 eV with that of PCDTBT (5.3 eV) donor material facilitates the hole transportation to ITO. The improved performance is also attributed to decreased R_s_ which is highly desired for carrier transportation and collection as evident from charge carriers mobility results. Moreover, the high R_sh_ of GO also helps to suppress carrier recombination. Both parameters were calculated from *J-V* curves. GO is probably inducing effective blocking of electron due to its large band gap of ~3.6 eV. In addition to the reasonably improved efficiency, reproducibility and stability, the preparation of HTLs as well as photoactive layer are based on a facile, flexible and R2R compatible solution process, which remarkably simplifies the overall fabrication process and lowers the fabrication cost.

## Additional Information

**How to cite this article**: Rafique, S. *et al*. Significantly improved photovoltaic performance in polymer bulk heterojunction solar cells with graphene oxide /PEDOT:PSS double decked hole transport layer. *Sci. Rep.* **7**, 39555; doi: 10.1038/srep39555 (2017).

**Publisher's note:** Springer Nature remains neutral with regard to jurisdictional claims in published maps and institutional affiliations.

## Figures and Tables

**Table 1:** Device photovoltaic performance characteristics of PCDTBT:PC_71_BM OPVs incorporating GO, PEDOT:PSS and GO/PEDOT:PSS as the HTLs.

Anode interlayer	J_sc_ (mA/cm^2^)	V_oc_ (V)	FF	Mobility, *μ* (cm^2^/Vs)	η (%)
GO	7.90	0.80	0.44	4.04 × 10^−4^	2.77
PEDOT:PSS	9.49	0.80	0.47	3.78 × 10^−4^	3.57
GO/PEDOT:PSS	10.44	0.82	0.50	7.47 × 10^−4^	4.28
